# Long-term treatment with lasmiditan in patients with migraine: post hoc analysis of treatment patterns and outcomes from the open-label extension of the CENTURION randomized trial

**DOI:** 10.1186/s10194-024-01745-y

**Published:** 2024-03-25

**Authors:** Mika Komori, Akichika Ozeki, Yuka Tanji, Eriko Kamiki, John H. Krege, Lily Qian Li, Shiho Suzuki, Mamoru Shibata, Takao Takeshima

**Affiliations:** 1 Japan Drug Development and Medical Affairs, Eli Lilly Japan K.K., 5-1-28, Isogamidori, Kobe-Shi, Chuo-Ku 651-0086 Japan; 2 Eli Lilly and Company, Indianapolis, USA; 3 Department of Neurology, Dokkyo Medical University, Mibu, Tochigi Japan; 4 Department of Neurology, Tokyo Dental College, Ichikawa General Hospital, Ichikawa, Japan; 5 Department of Neurology, Tominaga Hospital, Osaka, Japan

**Keywords:** Acute treatment, Lasmiditan, Migraine, Open-label

## Abstract

**Background:**

The objective of this analysis was to gain new insights into the patient characteristics and other factors associated with lasmiditan usage and clinical outcomes under conditions resembling the real-world setting.

**Methods:**

This was a post hoc analysis of data from the 12-month, open-label extension (OLE) of the phase 3, double-blind, randomized, controlled CENTURION trial, which examined the efficacy and safety of lasmiditan as acute treatment across four migraine attacks. Patients completing the main study who treated ≥ 3 attacks could continue in the OLE. The initial lasmiditan dose was 100 mg, with dose adjustments to 50 mg or 200 mg allowed at the investigator’s discretion. Patient and clinical characteristics were summarized by dosing pattern and completion status. Safety was assessed based on adverse event (AE) frequency by number of doses.

**Results:**

In total, 445 patients treated ≥ 1 migraine attacks with lasmiditan during the OLE, 321 of whom (72.1%) completed the study. Forty-seven percent of patients remained on the 100-mg initial dose during the OLE whereas 20.2% used both 100 mg and 50 mg, 30.6% used both 100 mg and 200 mg, and 6 (1.3%) used multiple dose levels. All dosing patterns were associated with clinical and patient-reported improvement; however, the 100-mg group had the highest proportion of patients reporting improvement in the Patient Global Impression of Change – Migraine Headache Condition (56.5% vs 33.4%–52.2%). In comparison, all three groups that made dose adjustments had higher rates of completion compared to the 100-mg group (72.1%–83.3% vs 68.9%). The frequency of AEs decreased with continued use of lasmiditan. Concomitant triptans and lasmiditan use did not increase AE frequency.

**Conclusions:**

Based on high persistence and patient satisfaction rates, the 100-mg dose appears optimal for most patients. For those who adjusted dose levels, dose adjustments appeared beneficial to improve efficacy or tolerability, retaining patients on treatment. Collectively, the data suggest that patients who experienced efficacy continued to use lasmiditan regardless of the occurrence or frequency of AEs, and continued use appeared associated with fewer AEs.

**Trial registration:**

European Union Drug Regulating Authorities Clinical Trials Database (EudraCT): 2018–001661-17; ClinicalTrials.gov: NCT03670810; registration date: September 12, 2018.

**Graphical Abstract:**

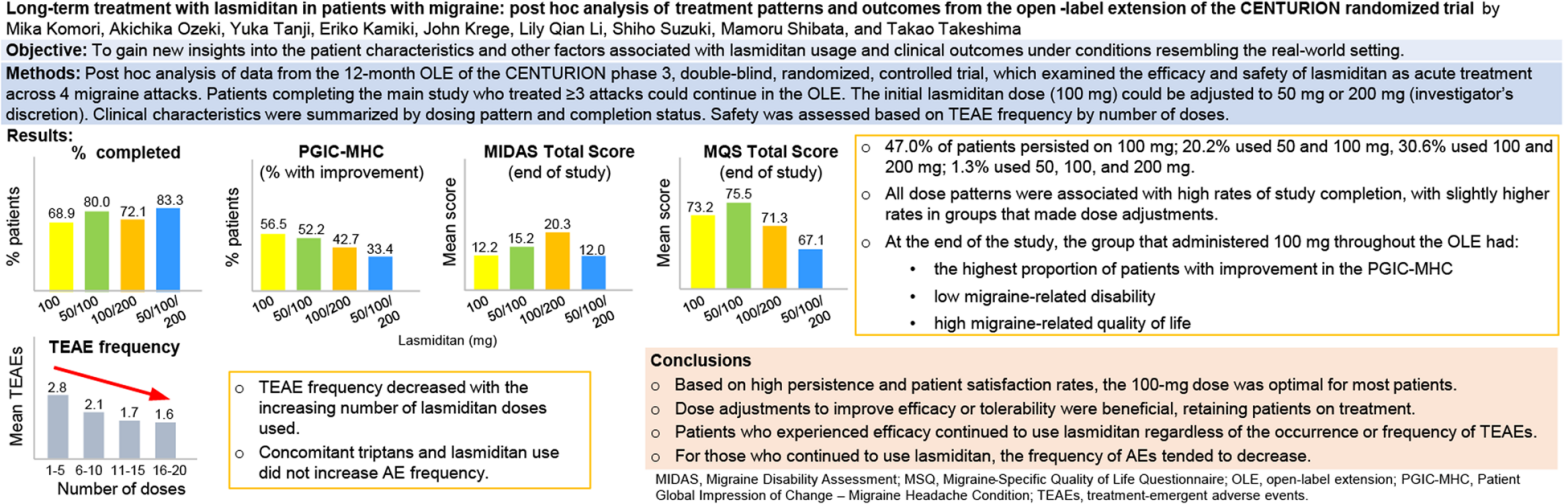

**Supplementary Information:**

The online version contains supplementary material available at 10.1186/s10194-024-01745-y.

## Background

Migraine is a debilitating primary headache disorder, negatively affecting both daily functioning and health-related quality of life. Globally, migraine is the second most common cause of disability among women aged 15 to 49 years, according to Global Burden of Disease 2019 data [1]. An analysis of global trends in the incidence of migraine from 1990 to 2019 indicated an increase of 40.1% to 87.6 million in this timeframe [2], with an estimated global prevalence of 14% [3]. Despite the significant global health burden of migraine, limited new therapeutic options for migraine have emerged in recent years, and there remains an unmet need for more effective and tolerable acute treatment regimens [4, 5].

Lasmiditan is a centrally penetrant, selective serotonin 1F receptor agonist (5-HT_1F_) and is the first approved drug in the novel ‘ditan’ class of medications. Lasmiditan was approved in October 2019 in the United States for the acute treatment of migraines with or without aura in adults and is now available in many countries [6, 7]. In studies of single-migraine attacks (SAMURAI, SPARTAN, and MONONOFU), lasmiditan demonstrated statistically significant superiority versus placebo in the proportion of patients who were pain-free as well as the proportion of patients who were free of their migraine-associated most bothersome symptom at 2 h postdose [8–10]. Lasmiditan additionally demonstrated efficacy and consistency of response across multiple attacks in the phase 3 CENTURION trial [11]. Lasmiditan is associated with generally mild or moderate central nervous system-related adverse events (AEs) of short duration; in multiple dose studies, these tend to decrease in frequency, with no increase in severity, across multiple attacks [12].

Following the CENTURION main study, a 12-month open-label extension (OLE) collected data on dose optimization, patterns of use, migraine-related disability, and quality of life during lasmiditan treatment. The OLE was designed to create conditions similar to those of a real-life treatment situation, with investigators permitted to adjust the lasmiditan dose and with patients not required to wait until pain became moderate-to-severe or to complete detailed electronic diary entries for each attack. Additionally, patients were permitted to take concomitant medications as needed [13]. During the OLE, there was a high rate of study completion, and patients reported improvements in migraine-related disability and quality of life, with no new safety findings observed after up to a year of treating attacks with lasmiditan [13].

However, as lasmiditan was relatively recently approved, several clinical questions remain surrounding its use in real-world clinical practice. The current analysis evaluated data from the CENTURION OLE to gain new insights into the patient characteristics and other factors associated with lasmiditan usage (such as dosing patterns and number of doses), study completions, AE frequency, and concomitant medication usage under conditions resembling the real-world setting.

## Methods

### Study design and patients

The CENTURION trial was a 4-month, multicenter, randomized, double-blind, placebo-controlled phase 3 study that examined lasmiditan efficacy and safety across 4 migraine attacks. The main study enrolled men and women from Asia, Europe, and North America aged ≥ 18 years with a history of migraine with or without aura for ≥ 1 year (International Classification of Headache Disorders-3 [14] classification 1.1 and/or 1.2.1 for migraine), 3 to 8 migraine attacks/month (< 15 headache days/month during the past 3 months), onset < 50 years, and disabling migraine defined as a Migraine Disability Assessment (MIDAS) score ≥ 11. The eligibility criteria and study design of the main study have been previously described in detail [11, 13].

Sixty-one sites in 12 countries in Europe and North America participated in the optional 12-month OLE of the CENTURION trial. Patients were eligible for the OLE if they treated ≥ 3 attacks during the main study and did not discontinue early, irrespective of their randomized treatment. The OLE study design has been previously described in detail [13] and is depicted in Additional file 1.

The study was carried out in accordance with the protocol, applicable local laws and regulations, and consensus principles derived from the International Conference on Harmonisation Good Clinical Practice guidelines, Declaration of Helsinki, and Council for International Organizations of Medical Sciences International Ethical Guidelines. The study protocol was approved by ethical review boards at each study site. Written informed consent was obtained from all patients prior to enrollment. The CENTURION trial is registered with the European Union Drug Regulating Authorities Clinical Trials Database (EudraCT: 2018–001661-17) and ClinicalTrials.gov (NCT03670810).

### Treatment and procedures

In the main study [11], patients were randomized 1:1:1 to lasmiditan 200 mg, lasmiditan 100 mg, or a control group that received placebo for three attacks and lasmiditan 50 mg for either the third or fourth attack. Patients were instructed to administer doses orally within 4 h of the onset of a moderate-to-severe migraine attack. Patients were considered to have completed the main study after treating four attacks or at 4 months after randomization.

In the OLE [13], patients were initially assigned to 100-mg lasmiditan, with flexible dosing (50, 100, or 200 mg) thereafter at the investigator’s discretion to optimize efficacy and tolerability. Patients were instructed to treat migraine attacks with lasmiditan when possible and were allowed to take lasmiditan for each new migraine attack provided a ≥ 24-h interval occurred between doses. If unable to treat with lasmiditan during a migraine attack, the patient was permitted to use their usual migraine medication for that attack. Patients were to refrain from driving, operating heavy machinery, or engaging in other similar activities for 8 h after taking the study drug.

### Assessments

Demographic and clinical characteristics of patients were examined by dosing pattern during the OLE period, based on the following cohorts: 1) patients who did not change the dose, continuing on the initial dose of 100 mg during the OLE; 2) patients who used both 50-mg and 100-mg dose levels during the OLE; 3) patients who used both 100-mg and 200-mg dose levels during the OLE; and 4) patients who used 50-mg, 100-mg, and 200-mg dose levels during the OLE. Patient characteristics were also examined in cohorts based on study completion status (completed; discontinued due to AE; discontinued due to lack of efficacy) and the number of doses administered.

The clinical characteristics summarized included the total number of lasmiditan doses administered, the average number of lasmiditan doses per month, the proportion of patients completing the study, the proportion of patients taking each of the treatments (placebo, lasmiditan 50 mg, 100 mg, or 200 mg) as their last dose in the double-blind study; the proportion of patients with each of the lasmiditan dose levels (50 mg, 100 mg, or 200 mg) as the modal dose (i.e., the dose administered most frequently); the proportion of patients with migraines with and without aura at visit 1 (screening), the number of migraine attacks per month in the last 3 months at visit 1 (screening); the proportion of patients who used a migraine preventive during the study, and the proportion of patients with prior triptan exposure.

Other clinical characteristics examined included data obtained from migraine assessment scales, which were assessed at specific visits, per the protocol. Data examined include migraine-related disability at visit 1 (double-blind study screening visit) and at each OLE visit as assessed by MIDAS total score, MIDAS headache days, and migraine severity [15, 16]. MIDAS was collected at double-blind study baseline and OLE study visits except visit 7 with a recall period of “in the past 3 months” and again at the end of study (visit 11) with a recall period of “since last visit”. For visit 6 (OLE baseline), to avoid an overlap in recall period, a weighted score was calculated based on the length of time a patient was enrolled in the study for total score and headache days: weighted MIDAS score = raw score × 90 days / days since baseline assessment.

Additional assessments included quality of life at visit 6 (OLE baseline) and visit 11 (end of study), as evaluated with the Migraine Specific Quality of Life Questionnaire (MSQ) version 2.1 total score and Role Restrictive, Role Preventive, and Emotional Function domain scores [17]. For the analysis of MSQ assessments by completion status, data from the early termination visit were analyzed for the last visit for those who discontinued. The 6-item Migraine Treatment Optimization Questionnaire (mTOQ-6) [18] score was examined at visit 2 (double-blind study baseline; current triptan users only), and the percentage of patients reporting each response on the Patient Global Impression of Change – Migraine Headache Condition (PGIC-MHC) [19] was examined at visit 11 (month 12; end of study).

Tolerability and safety were evaluated by assessment of treatment-emergent adverse events (TEAEs), defined as any AE with time of onset within 48 h after a dose of study drug, or any event that worsened in intensity within 48 h after a dose of study drug.

### Statistical analysis

The intent-to-treat (ITT) population included patients who received one or more doses of study drug during the OLE. Descriptive statistics were used for demographic and clinical characteristics, with continuous measures summarized by mean plus standard deviation (SD) or median (minimum, maximum) and categorical variables summarized by frequency counts and percentages.

A mixed model for repeated measures analysis was used to assess improvement in MIDAS total score (change from main study baseline) at each OLE visit. The total number of TEAEs was summarized by frequency, subcategorized by patient cohorts defined by the total number of doses administered during the study (≥ 10; ≥ 20). Mean differences in TEAE frequency by the number of administered lasmiditan doses in 5-dose intervals were analyzed using paired t-tests (parametric) and the Wilcoxon signed rank test (non-parametric), with a two-sided significance level of 0.05.

The number of times ergot alkaloids, nonsteroidal anti-inflammatory drugs (NSAIDs), or triptans were concomitantly administered with lasmiditan were summarized relative to the timing of lasmiditan administration. Additionally, the occurrence of TEAEs during migraine attacks co-treated with lasmiditan and triptans was summarized by frequency relative to the time of lasmiditan administration.

Statistical analysis was performed using SAS 9.4 (SAS Institute Inc., Cary, NC, USA) and R, Version 4.2.2 (R Foundation for Statistical Computing, Vienna, Austria) [20].

## Results

### Patient disposition

In total, 477 patients entered the OLE, and 445 patients treated one or more migraine attacks with lasmiditan during the OLE (ITT population). Of these, 321 patients (72.1%) completed the 12-month OLE study. Withdrawal by subject was the most frequent reason for discontinuing from the OLE (*n *= 38; 8.5%), followed by lack of efficacy (*n* = 36; 8.1%) and adverse event (*n* = 22; 4.9%).

### Lasmiditan dosing pattern during the OLE

In the ITT population, 209 (47.0%) patients remained on the 100-mg initial dose throughout the OLE whereas 90 (20.2%) patients used both 100-mg and 50-mg doses and 136 (30.6%) patients used both 100-mg and 200-mg doses. Additionally, 6 (1.3%) patients used multiple dose levels (50, 100, and 200 mg) of lasmiditan during the OLE.

Table 1 summarizes dose frequency in the dose pattern cohorts. Overall, the mean (SD) number of lasmiditan doses was 19.5 (17.09) in total and 1.7 (1.28) per month, and this did not substantially vary by dosing pattern in the OLE (Table 1). No relationship was apparent between the last dose in the double-blind study and the OLE dosing pattern (Table 1). For 52 of 90 patients (57.8%) in the 50-mg/100-mg group, 50 mg was the most frequently taken dose whereas for 93 of 136 patients (68.4%) in the 100-mg/200-mg group, 200 mg was the most frequently taken dose. A slightly higher proportion of patients in the 50-mg/100-mg (80.0%) and 50-mg/100-mg/200-mg groups (83.3%) completed the study compared with the continuous 100-mg group (68.9%) or the 100-mg/200-mg group (72.1%).

**Table 1:** Demographic and clinical characteristics for patient cohorts based on lasmiditan dosing pattern

Lasmiditan dosing summary^a^	100 mg (continuous) *n* = 209	50 mg/100 mg *n* = 90	100 mg/200 mg *n* = 136	50 mg/100 mg/ 200 mg *n* = 6	Total^b^ *N* = 445
**Total number of lasmiditan doses administered**	18.7 (18.08)	19.5 (16.42)	21.0 (16.28)	20.3 (8.71)	19.5 (17.09)
**Lasmiditan doses per month**	1.7 (1.34)	1.6 (1.22)	1.8 (1.23)	1.9 (1.41)	1.7 (1.28)
**Last dose in double-blind period,** *n* (%)^c^					
Placebo	35 (43.8)	14 (17.5)	28 (35.0)	1 (1.3)	80 (100)
50 mg	42 (48.3)	23 (26.4)	21 (24.1)	1 (1.1)	87 (100)
100 mg	66 (45.2)	28 (19.2)	50 (34.2)	2 (1.4)	146 (100)
200 mg	66 (50.0)	25 (18.9)	37 (28.0)	2 (1.5)	132 (100)
**Modal dose**^**d**^					
50 mg	0	52 (57.8)	0	3 (50.0)	57 (12.8)
100 mg	209 (100)	38 (42.2)	43 (31.6)	3 (50.0)	293 (65.8)
200 mg	0	0	93 (68.4)	0	94 (21.1)
**Completed, *n* (%)**	144 (68.9)	72 (80.0)	98 (72.1)	5 (83.3)	321 (72.1)
**Demographics and clinical characteristics**^**a**^	**100 mg (continuous) *n* = 209**	**50 mg/100 mg *n* = 90**	**100 mg/200 mg *n* = 136**	**50 mg/100 mg/ 200 mg *n* = 6**	**Total**^**b**^ ***N* = 445**
**Age,** years	41.5 (12.7)	41.1 (10.9)	45.3 (11.0)	42.5 (8.4)	42.5 (11.9)
**Sex, female,** n, (%)	181 (86.6)	82 (91.1)	112 (82.4)	5 (83.3)	383 (86.1)
**Body weight,** kg	76.5 (18.1)	74.9 (18.3)	81.0 (21.6)	79.0 (17.3)	77.5 (19.3)
**Migraine history, years**	16.1 (12.1)	19.5 (12.5)	21.2 (13.2)	14.8 (12.0)	18.3 (12.7)
**Migraine attacks/month in the 3 months prior to study baseline**	5.0 (1.6)	5.1 (1.5)	5.0 (1.5)	4.8 (1.5)	5.0 (1.5)
**Migraine, aura status, *n* (%)**					
With aura	45 (21.5)	28 (31.1)	24 (17.6)	0 (0.0)	98 (22.0)
Without aura	121 (57.9)	44 (48.9)	79 (58.1)	5 (83.3)	251 (56.4)
With and without aura	43 (20.6)	18 (20.0)	33 (24.3)	1 (16.7)	96 (21.6)
**Use of migraine preventive during study, *n* (%)**	67 (32.1)	29 (32.2)	41 (30.1)	2 (33.3)	140 (31.5)
**Triptan experience, *n* (%)**	133 (63.6)	64 (71.1)	97 (71.3)	6 (100.0)	302 (67.9)
**MIDAS Total Score**					
Baseline (V1)	25.8 (15.36)	31.4 (20.73)	30.4 (18.61)	18.8 (5.81)	28.4 (17.73)
Last visit (V11 EoS)	12.2 (15.04)	15.2 (14.88)	20.3 (31.51)	12.0 (6.28)	15.3 (21.64)
**MIDAS Headache Days**					
Baseline (V1)	15.7 (8.36)	15.8 (10.51)	18.2 (11.56)	17.8 (5.45)	16.5 (9.92)
Last visit (V11 EoS)	8.9 (9.40)	10.3 (10.12)	13.2 (14.91)	14.6 (23.92)	10.6 (11.90)
**MIDAS Average Pain Severity**					
Baseline (V1)	7.7 (1.54)	7.2 (1.74)	7.3 (1.74)	7.0 (2.24)	7.5 (1.67)
Last visit (V11 EoS)	6.1 (2.35)	6.1 (2.07)	6.4 (1.89)	5.6 (3.36)	6.2 (2.17)
**MSQ Total Score**					
OLE baseline (V6)	60.5 (17.54)	64.4 (13.58)	61.4 (16.37)	70.1 (10.58))	61.9 (16.31)
Last visit (V11 EoS)	73.2 (18.84)	75.5 (17.42)	71.3 (17.62)	67.1 (11.20)	73.2 (18.10)
**MSQ Role Function Restrictive**					
OLE baseline (V6)	54.1 (17.70)	57.6 (14.18)	54.7 (17.52)	60.1 (16.60)	55.3 (16.91)
Last visit (V11 EoS)	68.3 (20.61)	70.6 (18.25)	65.6 (19.13)	59.4 (14.48)	68.0 (19.65)
**MSQ Role Function Preventive**					
OLE baseline (V6)	67.5 (21.29)	71.6 (15.15)	68.6 (18.61)	88.0 (11.51)	69.1 (19.21)
Last visit (V11 EoS)	77.9 (18.75)	81.5 (16.99)	77.7 (17.20)	76.0 (18.84)	78.7 (17.89)
**MSQ Emotional Function**					
OLE baseline (V6)	66.9 (24.56)	70.7 (20.97)	67.4 (22.45)	69.3 (14.6`)	68.1 (23.01)
Last visit (V11 EoS)	78.4 (21.10)	79.0 (22.58)	76.3 (21.45)	73.3 (9.43)	77.9 (21.37)
**mTOQ-6** (V2 Baseline)^e^	4.8 (2.55)	4.6 (2.48)	4.3 (2.57)	4.8 (2.95)	4.6 (2.55)
**PGIC-MHC** *n* (%)					
Very much better	19 (9.1)	11 (12.2)	19 (14.0)	1 (16.7)	50 (11.2)
Much better	53 (25.4)	11 (12.2)	19 (14.0)	0	83 (18.7)
A little better	46 (22.0)	25 (27.8)	20 (14.7)	1 (16.7)	92 (20.7)
No change	58 (27.8)	33 (36.7)	59 (43.4)	4 (66.7)	157 (35.3)
A little worse	7 (3.4)	3 (3.3)	10 (7.4)	0	20 (4.5)
Much worse	1 (0.5)	1 (1.1)	2 (1.5)	0	4 (0.9)
Very much worse	0	1 (1.1)	0	0	1 (0.2)

*AE* adverse event, *EoS* end of study, *MIDAS* Migraine Disability Assessment, *MSQ* Migraine-Specific Quality of Life Questionnaire, *mTOQ-6* Migraine Treatment Optimization Questionnaire, *PGIC-MHC* Patient Global Impression of Change – Migraine Headache Condition, *V* visit

^a^Per study design, visits 1 and 2 represent baseline visits in the double-blind portion of the study whereas visit 6 corresponds to the OLE baseline visit. Mean (SD) shown unless otherwise indicated

^b^The total population includes 4 participants who did not administer 100 mg during the OLE and are not represented within any of the 4 cohorts. Data are summarized based on non-missing values at each corresponding time point

^c^The proportion of patients taking each of the treatments as their last dose in the double-blind study was calculated using the number of patients in each treatment group as the denominator

^d^For modal dose, the proportion of patients with each dose level as a modal dose was calculated using the number of patients in the dosing cohort as the denominator

^e^Data are for current triptan users only

The mean age, weight, and sex of the OLE patient population did not substantially differ by dosing pattern, although a slightly higher proportion of females were in the 50-mg/100-mg group whereas the 100-mg/200-mg group had a higher proportion of male and older patients (Table 1). Regardless of dosing pattern during the OLE, approximately one-third of all patients used a migraine preventive during the OLE. Additionally, approximately two-thirds had previous triptan experience (Table 1). The highest proportion of triptan-naïve patients (36.4%) were in the group who continued using 100 mg throughout the OLE (Table 1).

In the ITT population, MIDAS total scores, headache days, and average pain severity all decreased during the OLE (Additional file 2). When examined by dosing pattern during the OLE, the 50-mg/100-mg and 100-mg/200-mg groups had the highest mean MIDAS total scores at visit 1 (mean [SD] 31.4 [20.73] and 30.4 [18.61], respectively; Table 1). Mean MIDAS total scores were improved at visit 11 (month 12) in all cohorts; however, the 100-mg/200-mg group had the highest mean total score (mean [SD] 20.3 [31.51]), headache days (13.2 [14.91]), and average pain severity (6.4 [1.89]; Table 1) at the end of the study (visit 11).

In the ITT population, quality of life improved during the OLE, based on MSQ total and domain scores (Additional file 3). MSQ total scores at OLE baseline trended towards higher mean values in the 50-mg/100-mg and 50-mg/100-mg/200-mg groups (64.4–70.1) compared with the 100-mg continuous and 100-mg/200-mg groups (60.5–61.4), with similar trends observed for the MSQ domain scores (Table 1). At the end of study (visit 11), the mean MSQ total scores were higher in the 100-mg continuous group and 50-mg/100-mg group (73.2–75.5) compared with the other dosing groups (67.1–71.3), although the standard deviations were wide for mean MSQ scores (Table 1). At the end of study (visit 11), PGIC-MHC was highest in the cohort that continued using 100 mg throughout the OLE, with 56.5% of patients on this dosing regimen rating their migraines as ‘a little better’, ‘much better’, or ‘very much better’ compared with 33.4%–52.2% in the other dosing cohorts (Table 1).

### Continued and discontinued patient characteristics at baseline and postbaseline

Table 2 summarizes the characteristics of patients according to completion status and total doses administered. The mean (SD) total number of lasmiditan doses administered was 24.0 (17.45) for patients who completed the study compared with 6.2 (7.26) and 8.0 (7.30) for patients who discontinued the study due to AE or lack of efficacy, respectively. In total, 289 patients administered ≥ 10 lasmiditan doses during the study. The mean (SD) total number of lasmiditan doses was 4.3 (2.51) in the < 10 dose group and 27.7 (15.93) in the ≥ 10 dose group. The age and sex of the OLE patient population did not substantially differ by completion status or the number of doses administered (Table 2). Mean MIDAS total scores were lower at visit 1 in patients who completed the study (mean [SD] 28.4 [17.73]) compared with patients who discontinued due to AE (34.4 [34.67] or lack of efficacy (39.5 [33.32]). In addition, patients who completed the study had fewer headache days at study baseline (mean [SD] 16.5 [9.92]) compared with patients who discontinued due to AE (20.5 [8.53]) or lack of efficacy (18.9 [8.75]). Mean MIDAS total scores at study baseline did not differ by the number of doses administered, but at visit 11 (month 12), mean MIDAS total scores were higher in the group who administered ≥ 10 doses during the OLE (mean [SD] 17.2 [23.41]) compared with the group who administered < 10 doses (7.9 [9.18]; Table 2). In addition, patients who administered ≥ 10 doses had more headache days and greater average pain severity at baseline than those who administered < 10 doses (Table 2).

**Table 2:** Characteristics of dosing by study completion status and number of doses administered

**Completion status**				**Total number of lasmiditan doses**		**Total**^**b**^
**Lasmiditan dosing summary**^**a**^	**Completed** ***n* = 321**	**Discontinued due to AE** ***n* = 22**	**Discontinued due to lack of efficacy** ***n* = 36**	** < 10** ***n* = 156**	** ≥ 10** ***n* = 289**	***N* = 445**
**Total number of lasmiditan doses administered**	24.0 (17.45)	6.2 (7.26)	8.0 (7.30)	4.3 (2.51)	27.7 (15.93)	19.5 (17.09)
**Lasmiditan doses per month**	1.8 (1.34)	1.4 (0.80)	1.6 (1.04)	0.7 (0.56)	2.2 (1.24)	1.7 (1.28)
**Last dose in double-blind period,** *n* (%)^c^						
Placebo	53 (80.3)	5 (7.6)	8 (12.1)	40 (50.0)	40 (50.0)	80 (100)
50	61 (80.3)	6 (7.9)	9 (11.8)	30 (34.5)	57 (65.5)	87 (100)
100	106 (85.5)	5 (4.0)	13 (10.5)	47 (32.2)	99 (67.8)	146 (100)
200	101 (89.4)	6 (5.3)	6 (5.3)	39 (29.5)	93 (70.5)	132 (100)
**Modal dose,** *n* (%)^d^						
50 mg	52 (16.2)	0 (0)	3 (8.3)	11 (7.1)	46 (15.9)	57 (12.8)
100 mg	205 (63.9)	18 (81.8)	20 (55.6)	114 (73.1)	179 (61.9)	293 (65.8)
200 mg	64 (19.9)	4 (18.2)	13 (36.1)	30 (19.2)	64 (22.1)	94 (21.1)
	**Completion status**			**Lasmiditan doses**		**Total**^**b**^
**Demographics and clinical characteristics**^**a**^	**Completed** ***n*** **= 321**	**Discontinued due to AE** ***n*** **= 22**	**Discontinued due to lack of efficacy** ***n*** **= 36**	** < 10** ***n*** **= 156**	** ≥ 10** ***n*** **= 289**	***N*** **= 445**
**Age,** years	43.0 (12.0)	38.7 (10.3)	45.1 (9.9)	40.2 (11.8)	43.8 (11.7)	42.5 (11.9)
**Sex, female,** *n* (%)	271 (84.4)	19 (86.4)	33 (91.7)	136 (87.2)	247 (85.5)	383 (86.1)
**MIDAS Total Score**						
Baseline (V1)	28.4 (17.73)	34.4 (34.67)	39.5 (33.32)	25.5 (12.72)	29.1 (18.73)	28.4 (17.73)
Last Visit (V11 EoS)	15.3 (21.64)	NA	NA	7.9 (9.18)	17.2 (23.41)	15.3 (21.64)
**MIDAS Headache Days**						
Baseline (V1)	16.5 (9.92)	20.5 (8.53)	18.9 (8.75)	15.4 (7.97)	16.8 (10.34)	16.5 (9.92)
Last Visit (V11 EoS)	10.6 (11.90)	NA	NA	6.6 (9.01)	11.6 (12.34)	10.6 (11.90)
**MIDAS Average Pain Severity**						
Baseline (V1)	7.5 (1.67)	7.5 (1.62)	7.3 (1.40)	7.3 (1.73)	7.5 (1.65)	7.5 (1.67)
Last Visit (V11 EoS)	6.2 (2.17)	NA	NA	5.2 (2.72)	6.4 (1.95)	6.2 (2.17)
**MSQ Total Score**						
OLE baseline (V6)	61.9 (16.31)	59.1 (18.47)	58.3 (17.71)	64.6 (15.86)	61.2 (16.39)	61.9 (16.31)
Last Visit (V11 EoS or ET)^e^	73.2 (18.10)	66.9 (14.76)	58.8 (19.68)	81.7 (17.65)	70.9 (17.58)	73.2 (18.10)
**MSQ Role Function Restrictive**						
OLE baseline (V6)	55.3 (16.91)	52.9 (19.13)	50.6 (18.80)	57.8 (16.02)	54.6 (17.10)	55.3 (16.91)
Last Visit (V11 EoS or ET)^e^	68.0 (19.65)	59.7 (15.12)	52.2 (18.24)	77.8 (20.28)	65.5 (18.70)	68.0 (19.65)
**MSQ Role Function Preventive**						
OLE baseline (V6)	69.1 (19.21)	66.8 (20.08)	65.6 (18.39)	71.4 (19.64)	68.5 (19.09)	69.1 (19.21)
Last Visit (V11 EoS or ET)^e^	78.7 (17.89)	76.3 (13.83)	66.5 (23.05)	86.2 (15.18)	76.8 (18.06)	78.7 (17.89)
**MSQ Emotional Function**						
OLE baseline (V6)	68.1 (23.01)	63.2 (27.34)	66.5 (20.69)	71.6 (22.21)	67.2 (23.16)	68.1 (23.01)
Last Visit (V11 EoS or ET)^e^	77.9 (21.37)	71.2 (25.73)	63.7 (24.45)	84.8 (20.36)	76.2 (21.3)	77.9 (21.37)
**mTOQ-6** Baseline^f^ (V2)	4.5 (2.54)	5.0 (2.45)	4.0 (2.89)	4.6 (2.47)	4.6 (2.59)	4.6 (2.55)
**PGIC-MHC,** *n* (%) (V11 EoS)						
Very much better	46 (14.3)	1 (4.6)	0	15 (9.6)	35 (12.1)	50 (11.2)
Much better	77 (24.0)	0	0	18 (11.5)	65 (22.5)	83 (18.7)
A little better	83 (25.9)	3 (13.6)	1 (2.8)	23 (14.7)	69 (23.9)	92 (20.7)
No change	98 (30.5)	13 (59.1)	26 (72.2)	62 (39.7)	95 (32.9)	157 (35.3)
A little worse	10 (3.1)	3 (13.6)	4 (11.1)	11 (7.1)	9 (3.1)	20 (4.5)
Much worse	3 (0.9)	0	1 (2.8)	0	4 (1.4)	4 (0.9)
Very much worse	1 (0.3)	0	0	0	1 (0.4)	1 (0.2)

*AE* adverse event, *EoS* end of study, *ET* early termination, *MIDAS* Migraine Disability Assessment, *MSQ* Migraine-Specific Quality of Life Questionnaire, *mTOQ-6* Migraine Treatment Optimization Questionnaire, *NA* not available, *PGIC-MHC* Patient Global Impression of Change – Migraine Headache Condition, *V* visit

^a^Per study design, visits 1 and 2 represent baseline visits in the double-blind portion of the study whereas visit 6 corresponds to the OLE baseline visit. Mean (SD) shown unless otherwise indicated

^b^The total column pertains to the lasmiditan dose categories only, as not all reasons for discontinuation were studied herein under ‘completion status’. The total population includes 4 participants who did not administer 100 mg during the OLE. Data are summarized based on non-missing values at each corresponding time point

^c^The proportion of patients taking each of the treatments as their last dose in the double-blind study was calculated using the number of patients in each treatment group as the denominator

^d^For modal dose, the proportion of patients with each dose level as a modal dose was calculated using the number of patients in each completion status or dose number cohort as the denominator

^e^ET data shown for the last visit for patients who discontinued due to AE or lack of efficacy

^f^Data are for current triptan users only

Mean MSQ total scores at OLE baseline were similar across completion status groups (58.3–61.9) but were higher at the last visit in the group who completed the study (mean [SD] 73.2 [18.10]) compared with the groups who discontinued due to AE (66.9 [14.76]) or lack of efficacy (58.8 [19.68]). Mean MSQ total scores were also higher at both the OLE baseline and last visit for the group who administered < 10 doses of lasmiditan (visit 6: 64.6; visit 11: 81.7) compared with the group who administered ≥ 10 doses (visit 6: 61.2; visit 11: 70.9; Table 2). Similar trends across completion status and dose groups were observed for MSQ domain scores (Table 2).

For PGIC-MHC, the proportion of patients rating their migraines as ‘a little better’, ‘much better’, or ‘very much better’ at the end of study (visit 11) was lower in patients who discontinued due to AE (18.2%) or lack of efficacy (2.8%) compared with those who completed the study (64.2%; Table 2), and was also lower in patients who had administered < 10 doses of lasmiditan (35.8%) compared with patients who had administered ≥ 10 doses (58.5%).

### Safety

**Fig. 1:**
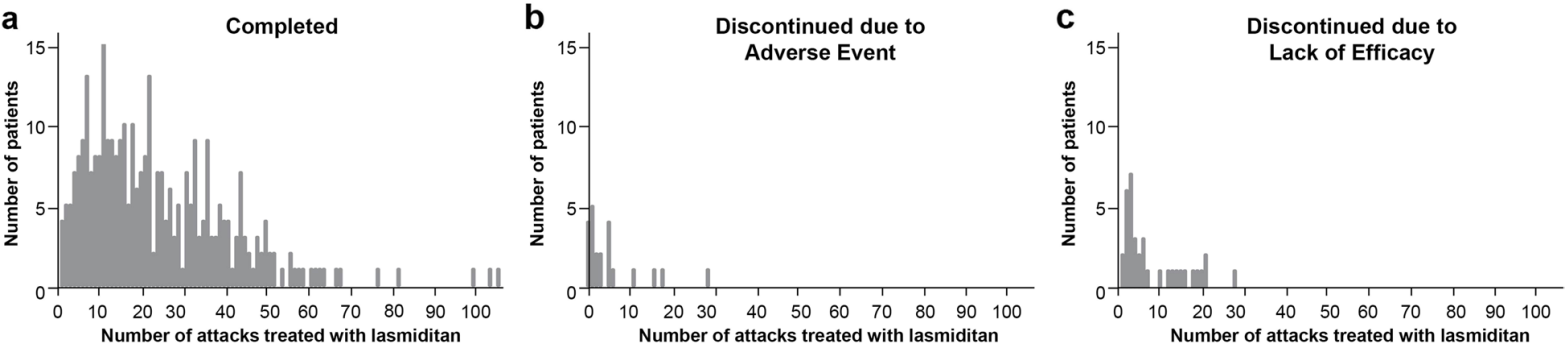
Histogram of the number of lasmiditan doses administered by completion status. **a** Completed; **b** Discontinued due to adverse event; and **c** Discontinued due to lack of efficacy

A histogram plot indicated that patients who discontinued early treated fewer attacks with lasmiditan than patients who completed the study. The majority of those who discontinued due to an AE took < 10 doses whereas the majority of those who discontinued due to lack of efficacy took < 20 doses (Fig. 1). To determine whether the frequency of TEAEs changed with continued lasmiditan use, mean differences in TEAE frequency were examined by the number of administered lasmiditan doses, assessed in 5-dose intervals. Among patients who administered ≥ 10 lasmiditan doses, the mean number of migraine attacks with TEAEs decreased by 0.758 from the first to fifth doses to the sixth to tenth dose of lasmiditan (*p* < 0.0001). For patients who administered ≥ 20 doses, the mean difference in the number of migraine attacks with TEAEs between the first to fifth dose and sixteenth to twentieth dose was 1.227 (*p* = 0.0001; Table 3).

**Table 3:** TEAEs by number of administered lasmiditan doses

***N***		**Number of doses**	**Mean number of TEAEs**^**a**^	**Mean difference**^**b**^	**95% confidence interval**	**Paired t-test**	**Wilcoxson signed rank test**
**TEAEs**							
Total doses ≥ 10	289	1st to 5th dose	2.86				
		6th to 10th dose	2.10	0.758	0.371, 1.145	*p* ≤ 0.0001	*p* < 0.0001
Total doses ≥ 20	181	1st to 5th dose	2.84				
		6th to 10th dose	2.07	0.768	0.226, 1.310	*p* = 0.0057	*p* = 0.0002
		11th to 15th dose	1.70	1.138	0.535, 1.741	*p* = 0.0003	*p* < 0.0001
		16th to 20th dose	1.61	1.227	0.613, 1.840	*p* = 0.0001	*p* < 0.0001
**Visits with TEAEs**			**Mean number of visits with TEAEs**	**Mean difference**^**a**^	**95% confidence interval**	**Paired t-test**	**Wilcoxson signed rank test**
Total doses ≥ 10	289	1st to 5th dose	1.61				
		6th to 10th dose	1.25	0.360	0.204, 0.516	*p* < 0.0001	*p* < 0.0001
Total doses ≥ 20	181	1st to 5th dose	1.59				
		6th to 10th dose	1.29	0.304	0.097, 0.511	*p* = 0.0042	*p* = 0.0018
		11th to 15th dose	1.12	0.475	0.241, 0.709	*p* < 0.0001	*p* < 0.0001
		16th to 20th dose	1.04	0.547	0.315, 0.779	*p* < 0.0001	*p* < 0.0001

N number of patients in analysis population, *n* number in category, *TEAEs* treatment-emergent adverse events

^a^Mean number of TEAEs per lasmiditan dose interval

^b^Mean difference from 1st to 5th dose values to post-baseline

### Concomitant medication usage

NSAIDs were the most commonly used concomitant medication in the OLE (Table 4), with 251 (2.9%) of lasmiditan-treated migraine attacks treated with NSAIDs up to 24 h prior to lasmiditan treatment. In addition, 96 (1.1%) attacks were treated with NSAIDs within 2-h post-lasmiditan treatment; and 471 (5.4%) were treated with NSAIDs between 2 and < 24 h following lasmiditan treatment. Triptans were the second most commonly used concomitant medication, with 112 (1.3%), 64 (0.7%), and 365 (4.2%) lasmiditan-treated attacks treated with triptans in the respective ≤ 24-h predose, < 2-h postdose, and 2- to < 24-h postdose categories.

**Table 4:** Summary of concomitant medication usage

Period of concomitant medication use relative to lasmiditan administration	Concomitant medication type	Number of times concomitantly administered *n* (%)^a^
From 24 h to immediately prior to lasmiditan administration	Ergot alkaloids	1 (0.0)
	NSAIDs	251 (2.9)
	Triptans	112 (1.3)
From 0 to < 2 h after lasmiditan administration	Ergot alkaloids	0
	NSAIDs	96 (1.1)
	Triptans	64 (0.7)
From 2 to < 24 h after lasmiditan administration	Ergot alkaloids	12 (0.1)
	NSAIDs	471 (5.4)
	Triptans	365 (4.2)

*N* number of migraine attacks treated with lasmiditan, *n* number of migraine attacks treated with lasmiditan in combination with other acute treatment, *NSAIDs* nonsteroidal anti-inflammatory drugs

^a^Percentages were calculated based on the number of times the concomitant medication was administered divided by the total number of times lasmiditan was administered (*N* = 8654)

Overall, 25.0% of treated migraine attacks in the OLE were associated with 1 or more TEAEs [13]. When triptan medication was taken up to 24 h prior to lasmiditan treatment, overall, 28.6% of attacks were associated with TEAEs whereas 31.3% and 37.5% of attacks were associated with TEAEs when triptans were taken within 2-h postdose and 2- to 24-h postdose, respectively (Table 5). Of these, the highest proportion of TEAEs associated with attacks occurred in the lasmiditan 100-mg group (33.3%–42.5% compared with 6.7%–28.2% and 20.0%–29.6% in the 50-mg and 200-mg groups, respectively; Table 5), but no consistent pattern in TEAE frequency emerged across dose levels in terms of the timing of triptan use relative to lasmiditan dosing. Lasmiditan taken with other concomitant acute migraine medications did not show any safety issues [13, 21].

**Table 5:** TEAEs associated with migraine attacks treated with lasmiditan in combination with triptans during the OLE

Period of triptan use relative to lasmiditan administration	Migraine attacks with TEAEs, *n* (%)^a^			
	**Lasmiditan 50 mg**	**Lasmiditan 100 mg**	**Lasmiditan 200 mg**	**Total**
From 24 h to immediately prior to lasmiditan administration	*n* = 10 2 (20.0)	*n* = 72 24 (33.3)	*n* = 30 6 (20.0)	*n* = 112 32 (28.6)
From 0 to < 2 h after lasmiditan administration	*n* = 15 1 (6.7)	*n* = 41 17 (41.5)	*n* = 8 2 (25.0)	*n* = 64 20 (31.3)
From 2 to < 24 h after lasmiditan administration	*n* = 39 11 (28.2)	*n* = 228 97 (42.5)	*n* = 98 29 (29.6)	*n* = 365 137 (37.5)

*N* number of migraine attacks treated with lasmiditan in combination with triptans, *n*, number of migraine attacks with events meeting specified criteria, *OLE* open-label extension, *TEAE* treatment-emergent adverse event

^a^Percentages were calculated based on the number of migraine attacks with TEAEs divided by the total number of migraine attacks that required the administration of triptans at each dose level. Lasmiditan dose groups correspond to the dose used concomitantly with the triptan to treat the attacks

## Discussion

This post hoc analysis of data from the 12-month OLE of the CENTURION trial was conducted to gain insight into lasmiditan use and safety under conditions resembling a real-world setting. The objective was to define clinical characteristics or patterns of use that were associated with outcomes from which new information could be distilled for prescribers to consider when treating patients with migraine. This analysis showed that the 100-mg dose appeared suitable as a starting dose for most patients, although dose adjustments appeared beneficial when applied, with no new safety concerns found. Our findings and their clinical implications are discussed in more detail below.

A high proportion of patients remained on the initial 100-mg dose throughout the OLE. Of those who temporarily or permanently changed dose levels, more patients increased their dose to 200 mg compared with those who decreased their dose to 50 mg. Few patients tried all three dose levels during the OLE. The pattern of lasmiditan dose adjustments was not strongly linked to patient demographics or migraine history nor did dosing pattern appear to impact lasmiditan usage in terms of doses administered in total or per month. A higher proportion of patients in the continuous 100-mg cohort reported improvement on the PGIC-MHC compared with the other dose cohorts. However, a slightly greater proportion of patients who adjusted their dose completed the study compared with those who did not. Of those who tried the other dose levels, 200 mg was the modal dose for two-thirds of the patients in the 100-mg/200-mg group, suggesting that the 200-mg dose was preferred by investigators and/or patients in this group during the OLE. An examination of the MIDAS data across the study indicates that patients in the 100-mg/200-mg group entered and ended the study with worse migraine disability compared with the total analysis population, which may suggest higher doses of lasmiditan were used to treat more severe cases. Nevertheless, the 100-mg/200-mg group showed similar relative improvement in disability at the end of the study to other dose groups and the proportion completing in this group was identical to the total analysis population. The 50-mg/100-mg group also showed improvement in migraine-related disability and had the second highest proportions of patients reporting improvement in the PGIC-MHC and completing the study. These data suggest that dose adjustments made to optimize efficacy or tolerability were associated with clinical improvement, patient-reported improvement, and a high rate of completion. Taken together with the finding that a higher proportion of patients who completed the OLE reported improvement compared with those who discontinued, the data collectively suggest that persisting with the 100-mg dose led to improvements for most patients while, for some, dose adjustments were beneficial in improving efficacy or tolerability and retaining patients on treatment. The current findings are consistent with those of a recent network meta-analysis of 12 clinical trials that examined the relative efficacy of lasmiditan (50, 100, and 200 mg) versus rimegepant (75 mg) and ubrogepant (25, 50, and 100 mg) as acute treatments for migraine. Lasmiditan 100 mg and 200 mg had greater efficacy at 2 h compared with either gepant whereas lasmiditan 50 mg showed similar efficacy to the gepants [22].

Mean MIDAS total scores and headache days were lower at the double-blind study baseline for those who completed the 12-month OLE compared with those who discontinued treatment, indicating that patients who went on to discontinue during the OLE had worse migraine disability at the beginning of the study. Additionally, mean MSQ total and domain scores at OLE baseline and last visit were higher in the group that completed the study compared with those who discontinued, indicating that migraine-related disability and quality of life across the study may have been contributing factors for discontinuing treatment.

Not unexpectedly, patients who discontinued due to an AE or lack of efficacy took fewer doses of lasmiditan in the OLE compared with those who completed the study (6.2–8.0 vs 24.0 doses). An examination of patient characteristics by maximum doses administered revealed that patients who administered 10 or more doses during the OLE had greater migraine disability and lower quality of life at the end of study compared with those who took fewer than 10 doses. Nevertheless, a higher proportion of patients in this group reported improvement in the PGIC-MHC compared with patients who took fewer than 10 doses in the OLE. Furthermore, for the groups who administered 10 or more doses and 20 or more doses of lasmiditan during the OLE, the frequency of TEAEs decreased with continued use of lasmiditan, consistent with previously reported results in the GLADIATOR trial and the double-blind portion of CENTURION [11, 23]. Collectively, the data may suggest that patients who experienced efficacy continued to use lasmiditan regardless of the occurrence or frequency of AEs. However, it should be noted that these results are from patients who were able to take multiple doses of lasmiditan during the OLE.

Lasmiditan is centrally penetrant and can cause central nervous system-related side effects [6]. As reported by Ashina et al., 2023 [13], during the OLE, the most common TEAEs were dizziness (35.7%), paraesthesia (16.2%), fatigue (14.6%), nausea (13.5%), vertigo (11.5%), somnolence (11.0%), and asthenia (5.8%), which were generally mild to moderate in severity. Flexible dosing and the permitted use of concomitant medications in the OLE confound the assessment of TEAEs by dose level; however, in the double-blind portion of the CENTURION trial, the 200-mg treatment arm reported a slightly higher frequency of common TEAEs during the first migraine attack compared with the 100-mg arm (e.g., dizziness, 26.5% versus 22.3%, respectively) [12]. The type and duration of common TEAEs in the main study were generally similar across migraine attacks, with the mean duration shortest for paraesthesia (less than 2 h) and ranging from 1.8 to 5.5 h for other common TEAEs [12]. The current safety analyses were in agreement with prior reports [11–13] and indicate no new safety concerns when lasmiditan was used in combination with other drugs, including triptans. Under study conditions resembling real-world use, NSAIDs and triptans were the most commonly used medications with lasmiditan. More patients chose to treat concomitantly with these medications after, rather than prior to, administering lasmiditan. Treating concomitantly with triptans after dosing with lasmiditan was associated with a slightly higher rate of TEAEs compared with treating with triptans prior to lasmiditan administration, but no consistent pattern in TEAE frequency emerged across dose levels in terms of the timing of triptan use relative to lasmiditan. Among those who treated concomitantly with triptans, TEAE frequency did not show a dose-dependent increasing trend, as the 200-mg group had a lower rate of TEAEs than the 100-mg lasmiditan group. Administration of triptans alone at commonly recommended starting doses has been shown to result in adverse events at rates of 16%–44% [24]. Therefore, the currently reported rates approximately at or below 40% for concomitant lasmiditan and triptan use are unlikely to have been due to the combination of drugs. In further support of this conclusion, a prior study reported that the type and frequency of common AEs were similar between patients who used lasmiditan alone and those who used lasmiditan concomitantly with triptans [21].

This analysis had limitations, which should be considered when interpreting the data. Notably, the flexible dosing in the OLE design meant a more heterogenous treatment regimen was followed, with fewer limitations on how patients treated their migraines. This better represented real-world clinical conditions than the double-blind portion of the study; however, the drawback of this design is that the range of responses on some assessments were broad, with large standard deviations making comparisons of the group means problematic. In addition, only 6 patients used multiple dose levels (50, 100, and 200 mg) of lasmiditan during the OLE and the number of patients discontinuing was low, limiting the conclusions that can be drawn from these groups. Finally, as the lasmiditan dose could be adjusted as needed to improve efficacy or tolerability during the OLE, it is difficult to determine the dose–response and exposure-safety relationships for lasmiditan during this period of the study.

## Conclusions

The OLE allowed dose optimization, the use of concomitant medication, and treatment of migraines prior to the pain becoming moderate-to-severe. Under these conditions, this analysis revealed that most patients persisted on 100-mg lasmiditan and reported the highest level of improvement in the PGIC-MHC at the end of the 12-month OLE. These results show that 100 mg is a suitable dose recommendation for most patients, in line with recommended doses in Europe and Japan [25, 26] and real-world usage in the United States [27]. For those who changed dose levels to optimize efficacy or tolerability, dose adjustments appeared beneficial and retained patients on treatment, resulting in slightly higher rates of completion than in the group that used 100 mg continuously in the OLE. These findings are of note as, in real-world clinical practice, lasmiditan administration may be discontinued early due to side effects without dose adjustment. Concomitant use of triptans did not synergistically increase AE frequency. Moreover, continued use of lasmiditan was associated with improved tolerability in terms of AE rates. Collectively, the data suggest that patients who experienced efficacy showed a high satisfaction level and continued to use lasmiditan, regardless of the occurrence of AEs, and for those who continued to use lasmiditan, the frequency of AEs tended to decrease.

### Supplementary Information

**Additional file 1.** Open-label extension study design schematic. The 12-month open-label extension (grey area) followed the double-blind section of the 4-month CENTURION study.

**Additional file 2.** Mean change from baseline in MIDAS Scores during the open-label extension; Table.

**Additional file 3.** Mean change from baseline in MSQ scores during the open-label.

## Data Availability

Eli Lilly and Company provides access to all individual participant data collected during the trial, after anonymization, with the exception of pharmacokinetic or genetic data. Data are available to request 6 months after the indication studied has been approved in the US and EU and after primary publication acceptance, whichever is later. No expiration date of data requests is currently set once data are made available. Access is provided after a proposal has been approved by an independent review committee identified for this purpose and after receipt of a signed data sharing agreement. Data and documents, including the study protocol, statistical analysis plan, clinical study report, and blank or annotated case report forms, will be provided in a secure data-sharing environment. For details on submitting a request, see the instructions provided at www.vivli.org.

## Abbreviations

5-HT_1F_: selective serotonin 1F receptor agonist
AE: adverse event
EoS: end of study
ET: early termination
ITT: intent-to-treat
MIDAS: Migraine Disability Assessment
MSQ: Migraine-Specific Quality of Life Questionnaire
mTOQ-6: Migraine Treatment Optimization Questionnaire
NSAIDs: nonsteroidal anti-inflammatory drugs
OLE: open-label extension
PGIC-MHC: Patient Global Impression of Change – Migraine Headache Condition
SD: standard deviation
TEAEs: treatment-emergent adverse events
V: Visit
