# Supplementary material for: Long-term treatment with lasmiditan in patients with migraine: post hoc analysis of treatment patterns and outcomes from the open-label extension of the CENTURION randomized trial
Source: J Headache Pain. 2024 Mar 25;25(1):43. doi: 10.1186/s10194-024-01745-y (PMC10964539; doi:10.1186/s10194-024-01745-y)
Supplement: Supplementary file 1 — Additional file 1. Open-label extension study design schematic. The 12-month open-label extension (grey area) followed the double-blind section of the 4-month CENTURION study. [file 10194_2024_1745_MOESM1_ESM.docx]

**
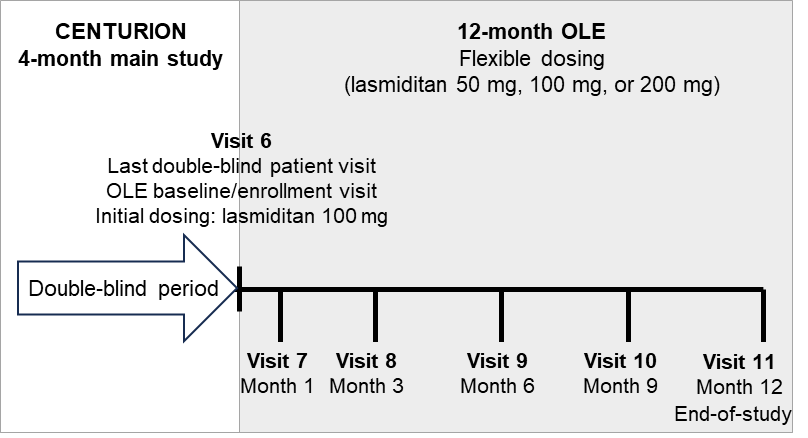
**

**Additional File 1.** Open-label extension study design schematic. The 12-month OLE (grey area) followed the double-blind section of the 4-month CENTURION study [13].

OLE, open-label extension.
