# Supplementary material for: Long-term treatment with lasmiditan in patients with migraine: post hoc analysis of treatment patterns and outcomes from the open-label extension of the CENTURION randomized trial
Source: J Headache Pain. 2024 Mar 25;25(1):43. doi: 10.1186/s10194-024-01745-y (PMC10964539; doi:10.1186/s10194-024-01745-y)
Supplement: Supplementary file 2 — Additional file 2. Mean change from baseline in MIDAS Scores during the open-label extension; Table. [file 10194_2024_1745_MOESM2_ESM.docx]

**Additional File 2. Mean change from baseline in MIDAS scores during the open-label extension**

|  | **Open-label lasmiditan (N=445)** | | | | | |
| --- | --- | --- | --- | --- | --- | --- |
|  | **MIDAS total score** | | **MIDAS headache days** | | **MIDAS average pain severity** | |
| **Visit** | **n** | **Mean change from baseline^a^** | **n** | **Mean change from baseline^a^** | **n** | **Mean change from baseline^a^** |
| **Visit 6/Month 0^b^** | 209^c^ | −13.3 (18.89) | 209^c^ | −7.1 (10.52) | 209^c^ | −0.4 (1.67) |
| **Visit 8/Month 3** | 324 | −11.7 (21.39) | 324 | −4.9 (11.82) | 324 | −0.8 (1.94) |
| **Visit 9/Month 6** | 307 | −12.0 (20.27) | 307 | −4.2 (11.98) | 307 | −1.0 (2.36) |
| **Visit 10/Month 9** | 310 | −13.5 (19.19) | 309 | −5.5 (12.76) | 309 | −1.1 (2.46) |
| **Visit 11/Month 12** | 303 | −13.0 (24.86) | 304 | −5.9 (14.06) | 304 | −1.3 (2.39) |

Mean (SD) shown.

^a^Baseline value was measured at visit 1 (screening visit) in the double-blind main study.

^b^Weighted scores were used for visit 6 total scores and headache days.

^c^As MIDAS quantifies headache-related disability over the previous 3-month period, data were incomplete for some patients at OLE baseline.

MIDAS, Migraine Disability Assessment; OLE, open-label extension; SD, standard deviation.
