# Supplementary material for: Long-term treatment with lasmiditan in patients with migraine: post hoc analysis of treatment patterns and outcomes from the open-label extension of the CENTURION randomized trial
Source: J Headache Pain. 2024 Mar 25;25(1):43. doi: 10.1186/s10194-024-01745-y (PMC10964539; doi:10.1186/s10194-024-01745-y)
Supplement: Supplementary file 3 — Additional file 3. Mean change from baseline in MSQ scores during the open-label. [file 10194_2024_1745_MOESM3_ESM.docx]

**Additional File 3. Mean change from baseline in MSQ scores during the open-label extension**

|  | **Open-label lasmiditan** | | | | | | | |
| --- | --- | --- | --- | --- | --- | --- | --- | --- |
|  | **MSQ Total Score** | | **Role Function Restrictive Score** | | **Role Function Preventive Score** | | **Emotional Function**  **Score** | |
| **OLE Visit** | **N** | **Mean** | **N** | **Mean** | **N** | **Mean** | **N** | **Mean** |
| **Visit 6/Baseline** | 437 | 61.6 (16.44) | 439 | 54.9 (17.07) | 438 | 69.0 (19.04) | 438 | 67.7 (22.71) |
|  | **n** | **Mean change from baseline** | **n** | **Mean change from baseline** | **n** | **Mean change from baseline** | **n** | **Mean change from baseline** |
| **Visit 7/Month 1** | 416 | 9.7 (16.66) | 418 | 10.8 (19.08) | 417 | 8.4 (18.62) | 417 | 9.1 (19.94) |
| **Visit 8/Month 3** | 382 | 6.9 (16.94) | 384 | 7.5 (18.70) | 383 | 6.4 (19.81) | 383 | 6.0 (20.44) |
| **Visit 9/Month 6** | 341 | 9.3 (17.84) | 344 | 10.5 (19.41) | 343 | 8.7 (20.56) | 342 | 7.7 (21.33) |
| **Visit 10/Month 9** | 322 | 11.2 (18.16) | 324 | 12.3 (19.54) | 324 | 10.1 (20.98) | 324 | 9.8 (21.85) |
| **Visit 11/Month 12** | 314 | 11.3 (19.40) | 316 | 12.7 (21.23) | 315 | 9.6 (21.56) | 315 | 9.8 (22.28) |
| **ET visit** | 93 | 4.8 (18.15) | 93 | 5.0 (19.65) | 93 | 5.5 (20.99) | 93 | 3.4 (21.37) |

Mean (SD) shown.

ET, early termination; MSQ, Migraine Specific Quality of Life Questionnaire; OLE, open-label extension; SD, standard deviation
